# Faster Insulin Aspart for Continuous Subcutaneous Insulin Infusion: Is It Worth It?

**DOI:** 10.7759/cureus.28422

**Published:** 2022-08-26

**Authors:** Patrícia Rosinha, Sofia Teixeira, Joana Vilaverde, Maria Helena Cardoso

**Affiliations:** 1 Endocrinology Department, Centro Hospitalar Baixo Vouga, Aveiro, PRT; 2 Endocrinology Department, Centro Hospitalar Universitário do Porto, Porto, PRT

**Keywords:** time below range, time above range, time in range, continuous glucose monitoring, type 1 diabetes mellitus, faster insulin aspart, continuous subcutaneous insulin infusion

## Abstract

Introduction

Faster insulin aspart (fASP) is the new formulation of insulin aspart (ASP) with a left-shifted pharmacokinetic profile, allowing better control of early postprandial hyperglycemia and a reduction in the risk of late post-meal hypoglycemia. However, it can be associated with more frequent infusion set changes. The purpose of this study is to evaluate efficacy and safety one, three, and six months after starting fASP in continuous subcutaneous insulin infusion (CSII) systems.

Methods

This is a retrospective study that included adults with type 1 diabetes mellitus, users of CSII ≥3 months, who started fASP. Exclusion criteria included less than one month of follow-up after the intervention, concomitant initiation of pharmacological therapy, pre-conception period, and non-use of continuous glucose monitoring.

Results

A total of 77 individuals were included, of which 52 (67.5%) were female, aged 39.87 ± 13.10 years, with a mean time under CSII of 7.30 ± 3.58 years and a median follow-up time after transition to fASP of six months. There was a trend to a global glycemic control improvement at six months after starting fASP: numeric increase in time in range (56.40 ± 12.62% vs 60.15 ± 13.53%, p=0.148), reduction in time above range (37.76 ± 13.05% vs 34.67 ± 14.94%, p=0.557), time below range (6.00 (5.00)% vs 4.50 (5.25)%, p=0.122), and mean glucose (174.29 ± 25.14 mg/dL vs 167.00 ± 25.30 mg/dL, p=0.207). There was a reduction in body mass index (BMI) at six months after switching to fASP (25.08 (4.59) kg/m^2 ^vs 24.45 (3.05) kg/m^2^, p=0.010), despite the absence of a significant variation in total daily insulin. Adverse event and discontinuation rates were 7.8% and 6.5%, respectively, with no documented episodes of diabetic ketoacidosis or severe hypoglycemia.

Conclusions

fASP proved to be a safe and effective therapeutic option in CSII systems associated with a significant BMI reduction, aspects that might justify its preference.

## Introduction

Type 1 diabetes mellitus (T1DM) is a condition in which the body is no longer able to produce adequate amounts of insulin as a result of the destruction of pancreatic beta cells, most often due to autoimmune cause, leading to increased blood glucose levels [1]. Both fasting and postprandial glucose values influence glycemic control, which translates in the risk of micro- and macrovascular complications [2]. The use of continuous glucose monitoring (CGM) in individuals with T1DM under continuous subcutaneous insulin infusion (CSII) therapy revealed that the control of postprandial glycemic excursions is still not achieved even after the transition from regular to rapid-acting insulin (RAI) analogs in CSII systems, creating the need for insulins with action profiles more similar to that of endogenous insulin [3]. It is now known that faster onset and offset of insulin action is desirable in individuals with T1DM to provide greater postprandial glycemic control, minimize hypoglycemic episodes and reduce weight gain [4,5].

The use of Fiasp® (Novo Nordisk A/S, Bagsværd, Denmark) in CSII systems was approved on September 30, 2019, in Portugal for individuals with T1DM. Faster insulin aspart (fASP) is the new formulation of insulin aspart (ASP) with two added excipients: niacinamide, which improves the absorption rate after subcutaneous injection by promoting vasodilation and formation of monomers, and L-arginine, which ensures formulation stability [6]. Studies show that the use of fASP in CSII translates into a left-shifted pharmacokinetic profile compared to ASP, meaning a three-fold higher early insulin exposure, an approximately 100% greater glucose-lowering effect at 30 minutes, and earlier offsets of glucose-lowering effect (24 minutes) and exposure (35 minutes) [5,7-9]. These pharmacokinetics and pharmacodynamics allow better control of early postprandial hyperglycemia and a reduction in the risk of late post-meal hypoglycemia compared to ASP. The randomized trial Onset 5 evaluated the efficacy and safety of fASP in CSII therapy over a clinically meaningful treatment period and confirmed the superiority of this insulin in the postprandial glycemic control and also the safety for CSII treatment [10]. Nonetheless, it also seems to be associated with more frequent infusion set changes due to local inflammatory reactions, which can be associated with a potentially higher risk of diabetic ketoacidosis in this population [11,12]. The purpose of this study is to evaluate the efficacy and safety at one, three, and six months after transitioning from RAI analogs to fASP in CSII systems.

The results of this study were previously presented as a meeting abstract at the 17^th^ Portuguese Diabetes Congress on March 11-17, 2021.

## Materials and methods

We conducted a retrospective observational study that included non-pregnant adults (≥18 years) with T1DM (for at least one year), using CSII therapy (MiniMed® Paradigm Veo, Medtronic plc, Dublin, Ireland, or Accu-Chek® Spirit Combo, Roche Diabetes Care, Basel, ‎Switzerland), who started fASP since this product was available in Portugal. All patients were previously treated with rapid-acting insulin (RAI) analogs. Exclusion criteria were: (a) less than one month of follow-up after insulin change, (b) concomitant initiation of any adjuvant pharmacological therapy, (c) pre-conception period, and (d) non-use of CGM. During the study period, a total of 87 individuals switched from RAI analogue to fASP but one patient had less than one month of follow-up after insulin change, four patients started concomitant sodium-glucose cotransporter 2 (SGLT2) inhibitors, four women were in the pre-conception period, and one patient did not use CGM. The study protocol was in conformance with the World Medical Association’s Helsinki Declaration and was approved by the Ethics Committee of Centro Hospitalar Universitário do Porto, Porto, Portugal (approval number: 2020.206(163-DEFI/164-CE)). Informed consent was waived by the Ethics Committee based on the retrospective nature of the study and full data anonymization. 

The study was conducted at Centro Hospitalar Universitário do Porto, Porto, Portugal. Data concerning demographic, anthropometric, glycemic control, and insulin requirements information was collected from the electronic clinical record at baseline and one, three, and six months after intervention (switch to fASP). The self-reported height was considered to calculate body mass index (BMI) as weight (kg)/height (m)^2^ at different times. Glycemic control was evaluated by CGM-obtained data of the last 28 days: mean glucose, time in range (TIR), time below range (TBR), and time above range (TAR). Data on daily carbohydrate intake, total daily insulin (TDI) dose, and total daily basal insulin (TDBI) dose in the last 30 days were downloaded from the CSII device software.

Safety was assessed by the number of episodes of diabetic ketoacidosis and severe hypoglycemia, defined by the American Diabetes Association as a severe event characterized by altered mental and/or physical status requiring assistance for treatment [13]. We also collected information regarding reported adverse events as well as fASP discontinuation rate.

Data analysis was performed using IBM SPSS Statistics for Windows, Version 20.0 (Released 2011; IBM Corp., Armonk, New York, United States). Categorical variables are presented as frequencies and percentages and continuous variables as means and standard deviations (SD) or medians and interquartile ranges (IQR) for variables with skewed distributions. Normal distribution was checked using the Shapiro-Wilk test or skewness and kurtosis as appropriate. All reported p-values are two-tailed, with a p<0.05 indicating statistical significance. Continuous variables were compared to baseline at different time points using paired-samples t-test or Wilcoxon test (if skewed distribution).

## Results

A total of 77 individuals were included, 52 (67.5%) females, at a current age of 39.87 ± 13.10 years, with a mean time under CSII of 7.30 ± 3.58 years and a median follow-up time after transition to fASP of six months (one to six months). The prevalence of overweight and obesity was 41.5% and 9.2%, respectively. Table 1 shows the baseline description of the sample with reference to the percentage of individuals treated with each of the RAI analogs and the prevalence of diabetes complications.

**Table 1:** Sample baseline characterization. ASP: aspart; CSII: continuous subcutaneous insulin infusion; GL: glulisine; LP: lispro; RAI: rapid-acting insulin; SD: standard deviation; T1DM: type 1 diabetes mellitus

Total, n (%)	77 (100.0)	
Male, n (%)	25 (32.5)	
Female, n (%)	52 (67.5)	
Age (years), mean ± SD	39.87 ± 13.10	
Time since diagnosis of T1DM (years), mean ± SD	22.92 ± 11.57	
Time under CSII (years), mean ± SD	7.30 ± 3.58	
Microvascular complications, n (%)	26 (33.8)	
Retinopathy	25 (32.5)	
Neuropathy	8 (10.4)	
Nephropathy	7 (9.1)	
Macrovascular complications, n (%)	2 (2.6)	
Previous RAI analog, n (%)		
LP	48 (62.3)	
ASP	24 (31.2)	
GL	6 (6.5)	

There was a reduction in BMI in all evaluated times after insulin change, but only with a statistically significant difference at six months after starting fASP (25.08 (4.59) kg/m^2^ vs 24.45 (3.05) kg/m^2^) (Table 2). There was a trend to a global glycemic control improvement at six months after starting fASP: numeric decrease in mean glucose observed at six months (174.29 ± 25.14 mg/dL vs 167.00 ± 25.30 mg/dL, p=0.207) and a progressive increase in TIR, with maximum at six months (56.40 ± 12.62% vs 60.15 ± 13.53%, p=0.148), in parallel with a reduction in TAR and TBR that were minimum at six months (37.76 ± 13.05% vs 34.67 ± 14.94%, p=0.557; 6.00 (5.00)% vs 4.50 (5.25)%, p=0.122) (Table 2). The TDI dose did not vary significantly in the evaluated times, nor its basal/bolus distribution (Table 2).

**Table 2:** Anthropometric parameters, glycemic control, and insulin requirements at baseline, and one, three, and six months after intervention. BMI: body mass index; IQR: interquartile range; M: month(s); SD: standard deviation; TAR: time above range; TBR: time below range; TDBI: total daily basal insulin; TDI: total daily insulin; TIR: time in range * Wilcoxon test; ^a^ paired-samples t-test

Variable	0M	1M	3M	6M	
Weight (kg), median (IQR)	69.10 (16.45)	69.00 (17.00)	66.00 (13.50)	67.00 (17.75)	
n	70	41	45	46	
p-value (vs baseline)*		0.365	0.220	0.006	
BMI (kg/m^2^), median (IQR)	25.08 (4.59)	25.20 (5.14)	24.44 (3.63)	24.45 (3.05)	
n	65	37	42	45	
p-value (vs baseline)*		0.151	0.561	0.010	
Glucose (mg/dL), mean ± SD	174.29 ± 25.14	168.62 ± 29.78	164.14 ± 26.77	167.00 ± 25.30	
n	65	43	60	63	
p value (vs baseline)^a^		0.471	0.934	0.207	
TIR (%), mean ± SD	56.40 ± 12.62	59.20 ± 15.03	59.98 ± 14.20	60.15 ± 13.53	
n	64	44	60	63	
p value (vs baseline)^a^		0.336	0.710	0.148	
TAR (%), mean ± SD	37.76 ± 13.05	35.10 ± 16.03	35.25 ± 13.93	34.67 ± 14.94	
n	64	44	60	62	
p-value (vs baseline)^a^		0.486	0.830	0.557	
TBR (%), median (IQR)	6.00 (5.00)	5.00 (5.75)	5.00 (5.00)	4.50 (5.25)	
n	64	44	60	62	
p-value (vs baseline)*		0.240	0.199	0.122	
Daily carbs intake (g), mean ± SD	148.17 ± 17.96	143.33 ± 24.44	133.50 ± 28.52	128.67 ± 24.58	
n	22	18	22	31	
p-value (vs baseline)^a^		0.791	0.232	0.924	
TDI dose (U/day), median (IQR)	42.25 (21.77)	39.70 (19.10)	40.70 (25.70)	41.60 (19.15)	
n	66	43	49	53	
p-value (vs baseline)*		0.382	0.655	0.099	
TDI dose (U/Kg/day), median (IQR)	0.59 (0.29)	0.55 (0.21)	0.68 (0.33)	0.60 (0.26)	
n	63	39	41	42	
p-value (vs baseline)*		0.948	0.946	0.754	
TDBI dose (U/day), median (IQR)	18.13 (10.00)	17.24 (8.06)	17.60 (10.43)	18.74 (8.68)	
n	66	43	48	52	
p-value (vs baseline)*		0.342	0.279	0.313	
TDBI dose (U/Kg/day), median (IQR)	0.25 (0.12)	0.25 (0.10)	0.29 (0.16)	0.28 (0.15)	
n	63	39	40	41	
p-value (vs baseline)*		0.513	0.109	0.762	

There were no documented episodes of diabetic ketoacidosis or severe hypoglycemia during follow-up. A total of six (7.8%) individuals reported adverse events related to the administration of fASP, most frequently pain (n=5, 6.5%) followed by hyperglycemia (n=1, 1.3%). A total of five (6.5%) individuals discontinued fASP: two during the first month (one for pain and one for hyperglycemia), two at three months (one for pain and the other for preference for the previous RAI analog), and one six months after intervention (for complaints of pain).

## Discussion

These results show that the use of fASP in CSII systems translated into a trend towards global glycemic control improvement at six months, with a numeric increase in TIR and a numeric reduction in TAR, TBR, and mean glucose.

Given the pharmacokinetic and pharmacodynamic profile of fASP in CSII systems and previous data published about fASP use in multiple injections users [14], the same or even a greater benefit in glycemic control would be expected after switching from RAI analog to fASP in CSII users. The retrospective nature of this study and the sample size are potential contributors to these results. However, these results can also be a reflex of inappropriate timing of bolus administration and the eventual need to adjust pump settings. 

Indeed, fASP is approved to be injected 0-2 minutes before a meal or 20 minutes after a meal and this indication is similar to RAI analogues. Nonetheless, it was already demonstrated that RAI analogues should be injected 15 to 20 minutes before a meal in order to effectively control postprandial glycemia. If we look to the pharmacokinetic and pharmacodynamic profile of fASP in CSII systems, fASP starts to act approximately 10 minutes earlier than ASP and this is why it seems reasonable that this insulin should be injected 5-10 minutes before a meal to maximize postprandial glycemic control, increase TIR, and simultaneously decrease TAR [5,6].

In the Onset 5 trial, we unexpectedly observed a trend of worsening glycemic control during the night and again the pharmacokinetic and pharmacodynamic characteristics of fASP with the earlier offsets of exposure and glucose-lowering effect were pointed at as the possible culprit. The eventual need to increase basal or use multiwave bolus in alternative to standard bolus were suggested as effective strategies to increase the efficacy of fASP in pumps [10]. In this study, there was neither a significant variation in the TDI dose nor in the basal/bolus distribution after switching to fASP and, in fact, this can be one of the explanations for our results. Due to its retrospective nature, it was not possible to evaluate both periods (day and night) separately, the type of bolus most used by patients, or if there was a change in the behavior of bolusing after the switch.

There was, on the other hand, a significant reduction in BMI at six months after the transition, despite the absence of significant variation in the TDI dose, basal/bolus distribution, or carbohydrate intake. The explanation for this outcome is not known but it appears an important result since weight gain associated with insulin treatment is both an undesirable side effect and a barrier to glycemic control. Since more than 50% of this cohort of patients were overweight or obese at baseline, this observed reduction in BMI with the switch to fASP takes an important place.

There were no documented episodes of diabetic ketoacidosis or severe hypoglycemia during follow-up. These results are reassuring because in both Onset 4 and Onset 5 trials there were higher rates of local reactions and more frequent infusion set changes, suggesting a higher potential to diabetic ketoacidosis in insulin pump users [10]. In this study, it was not possible to evaluate the rate of infusion set changes but the rate of discontinuation was about 6.5%, which is again a reassuring data. The adverse events reported were all mild and the rate was similar to the one observed in Onset 5 trial [10].

This real-world data study reinforces the benefits of using fASP in clinical practice, even though some limitations of our study should be noted. Firstly, its retrospective nature that contributed to the large number of missing values found in some variables and to the impossibility of collecting data related to the type of bolus used, timing of bolus administration, adjustment in pump settings, and quality of life metrics. Some of these missing values, namely the anthropometric parameters, were also related to the fact that part of the consultations were carried out by telemedicine due to the coronavirus disease 2019 (COVID-19) pandemic. Furthermore, it was not possible to include the value of glucose management indicator (GMI) because, in Portugal, both systems (manufacturer software and LibreView platform, Abbott Diabetes Care Inc., California, United States) are available to analyze the CGM-obtained data but they differ in the formula used to calculate GMI and the retrospective nature of this study did not allow to clarify which of the formulas was used in each case. Finally, the sample size might still have been insufficient to obtain statistically significant results in some variables (namely those obtained by CGM).

## Conclusions

In conclusion, these results show that the use of fASP in CSII systems translated into a trend towards global glycemic control improvement six months after the switch, with a numeric increase in TIR and a numeric reduction in TAR, TBR, and mean glucose. fASP has proved to be a safe and an equal effective therapeutic option in CSII systems compared to RAI analogs, with an additional benefit of BMI reduction, aspects that might justify the preference for this new insulin formulation.
